# Evaluation of a novel line probe assay to detect resistance to pyrazinamide, a key drug used for tuberculosis treatment^☆^

**DOI:** 10.1016/j.cmi.2017.05.026

**Published:** 2018-01

**Authors:** M. Driesen, Y. Kondo, B.C. de Jong, G. Torrea, S. Asnong, C. Desmaretz, K.S.M. Mostofa, S. Tahseen, M.G. Whitfield, D.M. Cirillo, P. Miotto, A.M. Cabibbe, L. Rigouts

**Affiliations:** 1) Mycobacteriology Unit, Institute of Tropical Medicine, Antwerp, Belgium; 2) R&D Laboratory, NIPRO Corporation, Shiga, Japan; 3) New York University, New York, USA; 4) Medical Research Council Unit, Fajara, Gambia; 5) National TB Reference Laboratory, Dhaka, Bangladesh; 6) National TB Reference Laboratory, Islamabad, Pakistan; 7) SA MRC Centre for Tuberculosis Research, DST/NRF Centre of Excellence for Biomedical Tuberculosis Research, Division of Molecular Biology and Human Genetics, Stellenbosch University, South Africa; 8) Emerging Bacterial Pathogens Unit, Division of Immunology, Transplantation and Infectious Diseases, IRCCS San Raffaele Scientific Institute, Milan, Italy; 9) Biomedical Sciences Department, Antwerp University, Antwerp, Belgium

**Keywords:** Line probe assay, *Mycobacterium tuberculosis*, *pncA*, Pyrazinamide, Resistance

## Abstract

**Objectives:**

The development of rapid molecular diagnostic assays for pyrazinamide (PZA) resistance is considered technically challenging as mutations are highly diverse, scattered along the full length of the *pncA* gene and not all are associated with PZA resistance. We evaluated the performance of the novel Genoscholar PZA-TB II line probe assay (PZA-LPA2; NIPRO Corporation, Japan).

**Methods:**

To evaluate the applicability of the PZA-LPA2 in clinical settings, we compared the performance of the PZA-LPA2 to a composite reference standard *pncA* Sanger and Illumina sequencing plus phenotypic susceptibility testing on a panel of 87 *Mycobacterium tuberculosis* isolates from World Health Organization (WHO) drug resistance surveys, harbouring mutations previously classified as associated or not associated with resistance according to data from peer-reviewed literature. In addition, the PZA-LPA2 was challenged against a selection of isolates with lineage-specific and non-resistance-associated mutations, for which the frequency among clinical isolates is unknown, and tested directly on 59 sputum extracts.

**Results:**

For the survey isolates, the PZA-LPA2 reached an overall agreement with the composite reference of 97.6% (80/82) or 94.3% (82/87) excluding or including heteroresistance, respectively. The PZA-LPA2 failed on 8.5% (5/59) of clinical samples; among valid results, 100% (14/14) sensitivity and 100% (7/7) specificity was reached relative to *pncA* Sanger sequencing.

**Conclusions:**

The PZA-LPA2 represents a valid and rapid alternative for indirect PZA susceptibility testing. Preliminary findings on clinical samples show promise for direct testing. Further studies are needed to assess the clinical risk of missing heteroresistance and falsely detecting lineage-specific, silent and nonassociated mutations.

## Introduction

Pyrazinamide (PZA) is a key sterilizing drug, which allowed for treatment shortening for drug-susceptible tuberculosis (TB) to 6 months. Most likely it is the only first-line drug to be maintained in all future TB regimens, including individualized and short (9 month) regimens for patients with multidrug-resistant TB [1], [2].

PZA is a prodrug that is converted to its active form, pyrazinoic acid, by pyrazinamidase [3], [4]. The main mechanism of PZA resistance in *Mycobacterium tuberculosis* (MTB) is the inactivation of pyrazinamidase as a result of mutations affecting the *pncA* gene encoding this enzyme. About 72–99% of PZA-resistant MTB strains have a mutation in *pncA* [5], [6]. The nonessential nature of the *pncA* gene allows the development of multiple mutations dispersed along the gene and promotor region without affecting bacterial fitness [7].

Phenotypic drug-susceptibility testing (DST) to PZA is currently not routinely performed in most drug-resistance surveys; nor is it performed in patient-oriented DST in most resource-limited settings, as the test is complicated by PZA requiring a precise acidic pH for its activity and suffers from limited reproducibility if not performed carefully [8], [9], [10], [11]. Molecular diagnostic–assays may bypass these difficulties and the infrastructural containment requirements for culture-based assays. The development of rapid molecular diagnostic assays for PZA resistance is considered technically challenging as mutations are highly diverse, scattered along the full length of the *pncA* gene and not equally associated with PZA resistance [6], [12], [13]. In addition, lineage-specific mutations in the *pncA* gene have been described for *Mycobacterium bovis, Mycobacterium canettii* and the *Mycobacterium tuberculosis* CAS/Delhi family [14].

The Genoscholar PZA-TB II assay (NIPRO Corporation, Japan; hereafter referred to as PZA-LPA2) is built on the line probe assay (LPA) principle and is the first commercial molecular test available for rapid detection of resistance to PZA. The PZA-LPA2 targets a 700 bp fragment designed to cover the entire *pncA* gene and putative promotor region up to nucleotide -18 of the wild-type MTB H37Rv reference strain and includes 48 probes, the majority of which represent the wild-type gene. Given the broad variety of *pncA* mutations associated with PZA resistance, no specific mutant probes are included, with absence of probe binding being considered as an indicator of PZA resistance. Importantly, the PZA-LPA2 also includes some probes representing mutations that are known not to confer PZA resistance, like the CAS/Delhi specific Ser65Ser, thus increasing the specificity of the assay.

In this study, we evaluated the performance of the PZA-LPA2 against a composite reference standard, including *pncA* sequencing and BACTEC Mycobacteria Growth Indicator Tube 960 (MGIT 960) system (Becton Dickinson, USA) testing at multiple concentrations. Our evaluation comprised primarily indirect testing on MTB isolates, with preliminary data directly on sputum specimens.

## Materials and Methods

### Clinical isolates

A panel of 87 MTB complex isolates obtained from national drug resistance surveys in Bangladesh and Pakistan and previously subjected to Illumina *pncA* sequencing and MGIT 960 PZA testing (Illumina, USA) [15], was studied by the PZA-LPA2. The isolates—representing all different mutations observed in these surveys—were selected and classified according to their likelihood to confer PZA resistance on the basis of previously published evidence [6], [12], [13] (Supplementary Table S1). The role of these mutations was assigned according to the phenotype observed. Ser65Ser mutants with an additional mutation associated with PZA resistance were classified on the basis of the additional mutation.

In addition to the survey isolates, the PZA-LPA2 was challenged against four lineage-specific isolates from the Belgian Co-ordinated Collections of Micro-organisms/Institute of Tropical Medicine (Antwerp) (BCCM/ITM) public collection and six non-resistance-associated mutants from South Africa [12] (Supplementary Table S2).

The total sample panel thus included 97 MTB isolates: 23 *pncA* wild-type, 22 isolates harboring mutations not associated with resistance (of which 14 Ser65Ser), 1 undetermined, 16 probably associated and 35 high confidence mutants (Table 1).

**Table 1:** PZA-LPA2 results vs. phenotypic DST stratified by mutation type

Characteristic	Susceptibility	PZA-LPA2 isolates from:						Total
		WHO survey			BCCM/ITM and South Africa			
*pncA* sequencing	MGIT 960	R	S	Subtotal	R	S	Subtotal	
High confidence	R	29^a^	3^b^	32	3		3	35
Probably associated	R	16^a^		16				16
Subtotal		45	3	48	3		3	51
Undetermined	S	1		1				1
Not associated	S	1	14	15	5		5	20
	R				2		2	2
WT	S		23	23				23
Subtotal		2	37	39	7		7	46
Total		47	40	87	10		10	97

BCCM/ITM, Belgian Co-ordinated Collections of Micro-organisms/Institute of Tropical Medicine (Antwerp); DST, drug-susceptibility testing; LPA2, Genoscholar PZA-TB II line probe assay; MGIT 960, BACTEC Mycobacteria Growth Indicator Tube 960; NT, not tested; PZA, pyrazinamide; R, resistant; S, sensitive; WHO, World Health Organization; WT, wild type.

a One of the isolates showed heteroresistance.

b All isolates showed heteroresistance.

### Clinical specimens

To collect preliminary information on the performance of the PZA-LPA2 on clinical samples, 59 smear-positive sputum samples were selected from routine service activities at the ITM [16] (Supplementary Table S3). PZA-LPA2 analysis was performed on the original DNA extract that was stored at less than −18°C since the PCR/sequence analysis (maximum storage was 14 months). The positive control included purified DNA from the H37Rv reference strain (BCCM/ITM 083715; 2 μL of 0.4 × 10^−3^ μg/μL).

### Phenotypic drug susceptibility

Determination of the minimal inhibitory concentration (MIC) was performed in MGIT 960 (Becton Dickinson, USA). To define the MIC for isolates from Bangladesh, a PZA stock solution (Acros Organics, Belgium; 15 000 μg/mL) was prepared in MGIT 960 PZA medium. By adding respectively 27.7, 55.3, 110.7 and 221.3 μL of stock solution to a MGIT 960 PZA tube, test concentrations of 50, 100 (critical test concentration), 200 and 400 μg/mL PZA were achieved. To define the MIC for isolates from Pakistan, the same final concentrations were used, yet a PZA stock solution was prepared by reconstituting 1 vial of lyophilized PZA (BACTEC MGIT 960 PZA kit) in dimethyl sulfoxide (33600 μg/mL), with subsequent serial dilutions in sterile distilled water and transfer of 100 μL of the respective working solutions (16800, 8400 and 4200 μg/mL) to the MGIT 960 PZA tubes. The H37Rv reference strain and ten EQA strains from the WHO project [15] were tested for each stock solution and did not reveal a difference in result between the different preparations. Isolates from South Africa had been previously tested by standard MGIT 960 PZA [12].

### DNA extraction

DNA was extracted from clinical isolates by transferring a 10 μL loop of bacilli grown on Löwenstein-Jensen medium to 400 μL of 1× TE buffer (10 mM Tris-HCl, 1 mM EDTA, pH 8.0) and boiling for 5 minutes at 100°C to release DNA and inactivate the material. DNA from sputum specimens was extracted by the Maxwell-LEV extraction kit (Promega, USA) or by the GenoLyse extraction kit (Hain LifeScience, Germany) as per the manufacturer's instruction (Supplementary Table S3).

### DNA sequencing

Targeted and whole genome sequencing were performed at the IRCCS San Raffaele Scientific Institute, Milan, Italy [15].

### Genoscholar PZA-TB II kit

Except for the DNA extraction, the PZA-LPA2 was run according to the manufacturer's instructions as specified in the kit insert, using the materials and reagents provided in the kit and the MULTIBLOT NS-4800 (NIPRO), providing a fully automated hybridization process. Reading of the strips was done manually. A positive control (H37Rv) was included in each run to facilitate the interpretation. Faint bands were considered absent if the band intensity was lower than the corresponding band on the positive control (Supplementary Fig. S1).

### Data analysis

To determine the precision of the PZA-LPA2 assay, one wild-type isolate and one *pncA* mutant were tested in triplicate (repeatability) by three operators (interoperator reproducibility) and on 3 days (day-to-day reproducibility). To calculate the overall performance on MTB isolates, the per cent agreement between the index test (PZA-LPA2) and composite reference standard (MGIT 960 PZA + *pncA* sequencing) was calculated as follows: ((*a* + *d*)/*n*) × 100, with *a* indicating true resistant samples, *d* true susceptible samples and *n* total number of samples. The performers and readers of the assays were blinded for clinical information and index test or reference standard results.

## Results

Overall, hybridization signals visualized as violet bands were strong, yet with slight variations in band intensity between isolates tested in the same run (Supplementary Fig. S2).

### Precision

All replicates from the precision experiments produced the expected banding profile, with clear bands, resulting in good intra- and interrun reproducibility.

### Performance for detection of *pncA* mutations and PZA susceptibility testing

A summary is provided in Table 1. Further details are in Supplementary Tables S1 and S2.

#### Survey isolates

The assay correctly identified 29 (90.6%) of 32 MGIT 960 PZA–resistant isolates with high confidence mutations. Similarly, all 16 probably associated mutants were resistant by the PZA-LPA2 and MGIT 960. The strain with an undetermined mutation (Thr153Pro) showed a very weak band at probe 37 that covers codons 149 to 154—and hence was scored resistant—while it was susceptible by MGIT 960. Given the lack of literature data on this mutation and our susceptible result in MGIT 960, we classify this as a false-resistant LPA result.

The false-susceptible LPA results for three isolates harboring high confidence mutations were likely due to the presence of heteroresistance, as evidenced by targeted sequencing, while two other isolates—showing a mixed pattern by targeted sequencing—were found to be resistant by the PZA-LPA2. The LPA result was not clearly linked to the proportion of mutants observed, ranging 15–42% among the three isolates with a wild-type LPA profile and 30–54% among the two LPA mutants (Supplementary Table S4).

All 23 *pncA* wild-type isolates and all 14 lineage-specific c195t (Ser65Ser) mutants were correctly identified as wild type by the PZA-LPA2 and yielded a susceptible MGIT 960 PZA result. The isolate harbouring another mutation not associated with resistance (insertion of nucleotide c after position -3 of the promotor region) and was MGIT 960 susceptible in our evaluation but showed genotypic resistance in the PZA-LPA2. This result is considered a false-resistant sample on the PZA-LPA2.

For the representative sample in the context of formal WHO drug resistance surveys, the PZA-LPA2 reached an overall agreement with the composite reference for 97.6% (80/82) when excluding the five heteroresistant isolates, while inclusion of all isolates resulted in an overall agreement of 94.3% (82/87); a specificity of 94.9% (37/39) was achieved.

#### BCCM/ITM isolates and isolates from South Africa

The five isolates from South Africa with point mutations not associated with resistance—including the synonymous mutation Lys96Lys—and previously identified as MGIT 960 PZA susceptible yielded a false-resistant PZA-LPA2 result, while the high confidence isolate with large deletion, showing an absence of wild-type bands 31–48, was found resistant by MGIT 960 and the PZA-LPA2. The two *M. bovis* isolates with a His57Asp high confidence mutation and the two *M. canettii* isolates with Ala46Ala silent mutations in *pncA* were found to be resistant by both MGIT 960 PZA and PZA-LPA2. The intrinsic PZA resistance of *M. canettii* might be due to mutations in *rpsA* or *panD* [17], i.e. not included in the PZA-LPA2. Thus, the phenotypic and genotypic tests were concordant despite the PZA-LPA2 identifying a silent mutation rather than the resistance conferring mutation or mutations outside of *pncA.*

The same analysis on all isolates (not restricted to the clinically more relevant selection of survey isolates) would result in respectively 92.4% (85/92) and 89.7% (87/97) agreement and specificity of 84.8% (39/46) for this albeit highly selected isolate panel.

### Preliminary evaluation of PZA-LPA2 on clinical specimens

All 59 sputum samples yielded a valid result on the basis of the positive amplification control, but four of them had a negative *pncA* band and one of them showed overall weaker bands, invalidating the final result, yielding a 91.5% success rate. Three of the five samples with invalid results were extracted by the GenoLyse kit and two with the Maxwell-LEV extraction kit, while 53 of 54 samples with valid results were extracted by the Maxwell-LEV extraction kit.

Among the remaining 54 sputum samples with valid results, accuracy was calculated for 21 samples with available *pncA* sequences. All mutants (*n* = 14) and wild types (*n* = 7) were correctly identified.

## Discussion

The PZA-LPA2 performed well in detecting *pncA* mutants in positive cultures, approaching the performance of currently endorsed molecular assays for detection of resistance to rifampicin [18], [19]. In the WHO survey context, PZA-LPA2 reached an excellent overall agreement with the composite reference standard of 97.6% or 94.3%, with exclusion or inclusion of heteroresistant isolates respectively.

The assay may, however, have some limitations.

For a first evaluation of the assay performance, we included samples from drug resistance surveys in Pakistan and Bangladesh, reflecting a realistic representativeness of *pncA* mutation patterns in these clinical settings, yet not exhaustively all possible *pncA* mutants worldwide. Hence, specificity and sensitivity were calculated for these specific settings and may vary geographically, thus stressing the necessity of larger multicentre studies.

A ‘no wild-type’ approach will not be 100% accurate because we expect a small percentage of mutations not causing resistance to be detected, which could be overcome through a diagnostic algorithm requiring confirmatory *pncA* sequencing for resistant LPA profiles.

The frequency of these mutations among clinical isolates, however, remains largely unknown and in the literature is limited to only one or two isolates per mutation in most cases. Also, the association of these mutations with phenotypic PZA susceptibility remains ambiguous, with contradictory findings on small numbers of tested isolates [6], [12], [13]. In the Bangladesh and Pakistan survey, only two such mutants were identified: the previously described insertion ‘c’ at position -3 of the promotor region, and the newly identified Thr153Pro. Inclusion of selected mutants from South Africa intentionally overrepresented these rare mutations in the study sample [6], even though our study only comprised three of ten mutations described by Whitfield et al. [12]. Nevertheless, the ‘no wild-type’ approach is also applied to interpretation of the *pncA* target sequence, which in turn correlates strongly with phenotypic DST testing in qualified laboratories. Should additional phylogenetic markers be discovered, the manufacturer would have the possibility to exclude these in future versions of this LPA. Besides—depending on the regional context—diagnostic algorithms can be adjusted with confirmatory sequencing of the *pncA* gene. A whole-genome sequencing approach combined with MIC testing will further clarify the phenotypic–genotypic association [20].

Given its design—the absence of wild-type probes considered to be mutation without confirmatory mutant probes—detection of heteroresistance is incomplete for the PZA-LPA2. The clinical significance of PZA heteroresistance is not fully understood and its frequency not systematically documented, yet it seems minimal for this target (A. M. Cabibbe, unpublished data).

In addition, mutations in *rpsA* and *panD* were found to be involved in PZA resistance [6] yet are not covered by the PZA-LPA2. Their prevalence and contribution to clinically relevant PZA resistance is presently unknown.

Even though the strips are relatively small and contain numerous probes, the interpretation was considered to be straightforward by the operators. Moreover, despite the slight variations in band intensity observed among different isolates tested during the same run, the automated hybridization assay showed high intra- and interrun reproducibility, with no apparent operator dependence, and replicates produced identical banding profiles.

The PZA-LPA2 could be relatively easily implemented in high multidrug-resistant TB prevalence settings—where testing is most needed [15]—as the LPA technology is already widely established at the central or intermediate level. From this perspective, we compared the Twincubator (Hain LifeScience) and the MULTIBLOT NS-4800 (NIPRO) as a hybridizing platform on ten isolates. Our preliminary data showed two false-susceptible and one false-resistant result for the Twincubator using nonoptimized test conditions (data not shown). Further optimization is ongoing by NIPRO, with promising preliminary results (personal communication). This would allow interchangeability of platforms enhancing the rollout and accessibility of this assay.

Finally, the highest added value of molecular tests over phenotypic DST is its direct applicability to clinical specimens, bypassing the need for biosafety containment facilities. Our study—even though on a small sample—shows promise for this application, although it suggests further optimization may be warranted to decrease the proportion of invalid results (17.4%, 4/23). Nine studies reporting on indeterminate results for the Genotype MTBDR*sl* (Hain Lifescience, Germany) showed a mean of 7.1% (147/2059) [19].

In conclusion, the PZA-LPA2 offers a valid molecular alternative for rapid indirect PZA susceptibility testing, with further evaluation needed on clinical specimens to consolidate our preliminary findings.

## Figures and Tables

**Figure:**
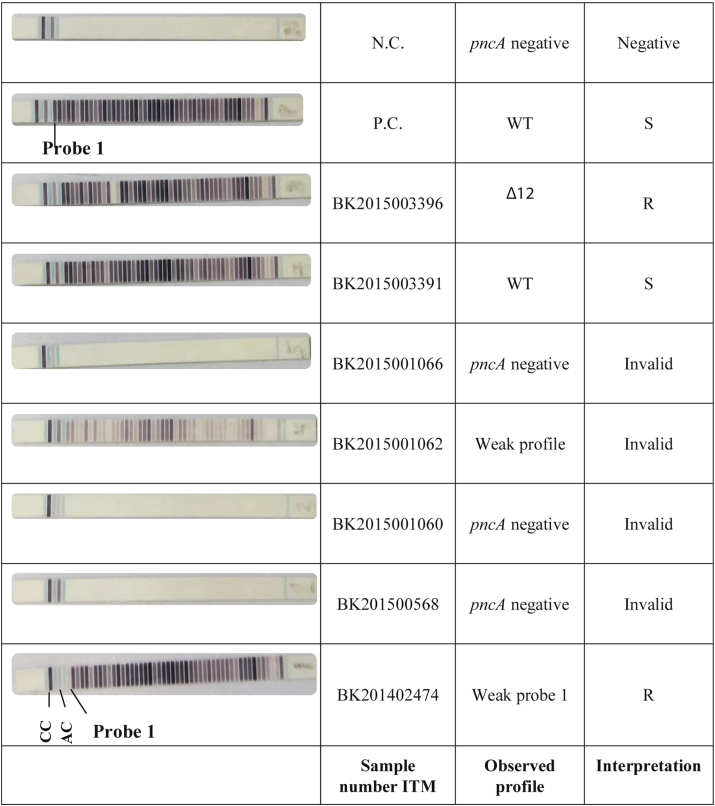


**Figure:**
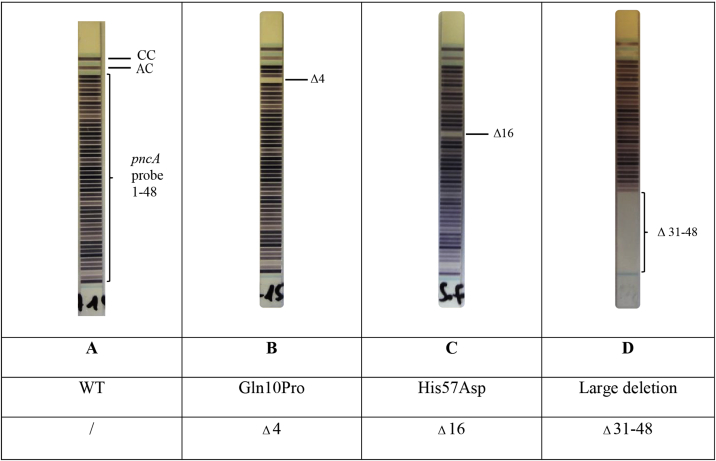


## Transparency Declaration

LR and BdJ were supported by European Research Council starting grant INTERRUPTB (311725). Clinical specimens were collected from studies funded by the Union, the Damien Foundation and the Belgian Directorate General for Development. Research reported in this publication was supported by the South African Medical Research Council. The content is the solely the responsibility of the authors and does not necessarily represent the official views of the South African Medical Research Council. MD reports nonfinancial support from NIPRO, Japan, during the conduct of the study. The other authors report no conflicts of interest relevant to this article.

## Appendix A Supplementary data

Title: PZA-LPA2 on clinical specimens

Description: Isolates showing one or more faint bands (as compared to the amplification control) at the position of the mutated codon (e.g. BK201402474) were considered resistant, given that the band intensity was lower than the corresponding band on the positive control included in the same run. CC: coloring control; AC: amplification control; P.C.: positive control; N.C.: negative control

Title: Examples of PZA-LPA2 strips

Description:CC: coloring control; AC: amplification control; A: WT; B: Gln10Pro *pncA* mutant; C: His57Asp mutant; D: large deletion in *pncA*
